# Metabolomic Characterization of Cerebrospinal Fluid from Intracranial Bacterial Infection Pediatric Patients: A Pilot Study

**DOI:** 10.3390/molecules26226871

**Published:** 2021-11-15

**Authors:** Yiwen Wang, Yu Liu, Ruoping Chen, Liang Qiao

**Affiliations:** 1 Department of Chemistry, Shanghai Stomatological Hospital, Fudan University, Shanghai 200000, China; 16307110373@fudan.edu.cn; 2 Department of Neurosurgery, Shanghai Children’s Hospital, Shanghai Jiao Tong University, Shanghai 200062, China; liuyu@shchildren.com.cn; 3 Department of Pediatric Neurosurgery, Xinhua Hospital, Shanghai Jiao Tong University School of Medicine, Shanghai 200092, China

**Keywords:** cerebrospinal fluid, central neural system infection, mass spectrometry, metabolomics, metabolic pathway

## Abstract

Intracranial bacterial infection remains a major cause of morbidity and mortality in neurosurgical cases. Metabolomic profiling of cerebrospinal fluid (CSF) holds great promise to gain insights into the pathogenesis of central neural system (CNS) bacterial infections. In this pilot study, we analyzed the metabolites in CSF of CNS infection patients and controls in a pseudo-targeted manner, aiming at elucidating the metabolic dysregulation in response to postoperative intracranial bacterial infection of pediatric cases. Untargeted analysis uncovered 597 metabolites, and screened out 206 differential metabolites in case of infection. Targeted verification and pathway analysis filtered out the glycolysis, amino acids metabolism and purine metabolism pathways as potential pathological pathways. These perturbed pathways are involved in the infection-induced oxidative stress and immune response. Characterization of the infection-induced metabolic changes can provide robust biomarkers of CNS bacterial infection for clinical diagnosis, novel pathways for pathological investigation, and new targets for treatment.

## 1. Introduction

Cerebrospinal fluid (CSF), the biofluid circulating from the choroid plexus toward the lumbar sac and venous blood, carries abundant information regarding the metabolic and transport state of the central neural system (CNS) [1,2]. Compared with plasma, CSF has a low protein concentration and simple metabolome [3]. Multi-omics characterization of CSF has curated a sum of ~250 miRNAs, ~3400 proteins, ~900 peptides and ~440 metabolites [4,5,6,7,8]. The multi-omics profiling of cerebrospinal fluid (CSF) holds great promise to gain insights into CNS diseases, including neurodegeneration [9], brain tumor [10], brain injury [11], and CNS infection [12].

The CNS is endowed with the blood–brain barrier and alloantigen tolerance to maintain its homeostasis [13]. Pathogens, however, can still invade the CNS from blood, surrounding tissues or nerves [14]. Intracranial surgery, on the other hand, can severely disrupt the physiological and immunological barriers in the CNS, leaving the CNS susceptible to various pathogens [15]. Nowadays, intracranial bacterial infection remains a major cause of morbidity and mortality in neurosurgical cases [16]. To elucidate the mechanism of the pathogenesis, multi-platform characterization of CSF from intracranial bacterial infection patients has been performed. NMR and MS-based methods offer high-throughput profiling, while ELISA and various biochemical reagent kits provide orthogonal quantification of certain biomolecules [17]. Bakochi et al. mapped the CSF proteome and depicted the dysregulation of 79 host proteins, suggesting an increase in apoptosis and glial cell development in response to acute bacterial meningitis [18]. Laarhoven et al. measured the metabolome of CSF from patients with tuberculous meningitis using a liquid chromatography mass spectrometry (LC-MS) platform and suggested tryptophan as a predictor of survival [19]. Quist-Paulsen et al. further profiled CSF metabolites in the tryptophan metabolism pathway and cytokines across patients with CNS bacterial infection and autoimmune neuroinflammation, and reported an IFN-γ mediated up-regulation in tryptophan metabolism in case of bacterial meningitis [20]. Moreover, the inflammatory CNS profile was different between pediatric cases and adult patients, including a lower elevated level of protein and white blood count, and a higher elevated level of aspartate transaminase in pediatric cases [21].

Metabolomics, by characterizing metabolites and metabolism, can directly and dynamically reflect pathophysiological phenotypes, thus demonstrating great potential in biomarker discovery and mechanism elucidation [22,23,24,25]. Untargeted and targeted metabolomics represent two differed pursuits of broad coverage and reliable quantification [26]. The combination of untargeted and targeted strategy, therefore, offers both global discovery and reliable verification of potential biomarkers of CNS infection [27]. Several perturbed pathways in response to CNS infection have been reported, including glycolysis, amino acids metabolism, and creatine biodegradation. [19,28,29,30,31]. Additionally, several metabolites have been proposed to be involved in the infection-induced osmosis stress (betaine and glutamate) and oxidative stress (urate, glutathione, ascorbate and carnitine) [31,32,33,34,35]. Nevertheless, there is still a lack of systematic characterization of CSF for the study of CNS infection to further discover and verify novel pathological pathways. Especially, there lacks understanding of the metabolomic changes in pediatric patients with CNS infection.

In this study, both untargeted and targeted analysis were performed for the characterization and measurement of CSF metabolome, to investigate the metabolic dysregulation in response to postoperative intracranial bacterial infection of pediatric cases. We included eight infection and six control CSF samples for metabolomic analysis as a pilot study, found 206 differential metabolites out of the 597 metabolites identified, and verified the glycolysis, amino acids metabolism, and purine metabolism pathways as potential pathological pathways. Characterization of the infection-induced metabolic changes can provide robust biomarkers of CNS bacterial infection for clinical diagnosis, novel pathways for pathological investigation, and new targets for treatment.

## 2. Results and Discussions

**Scheme 1:**
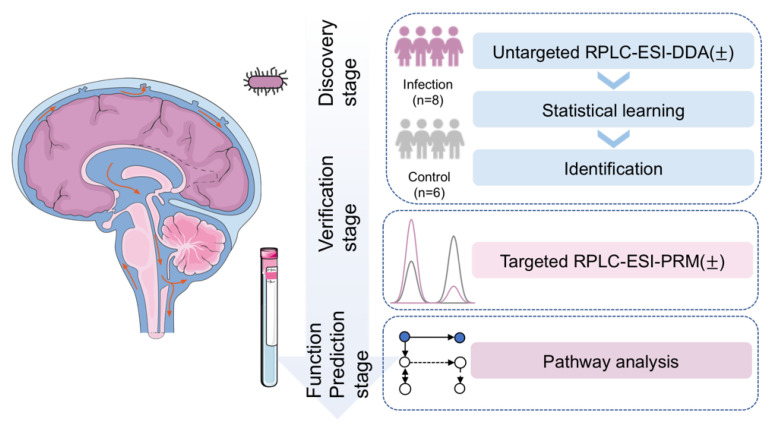
The pseudo-targeted metabolomic workflow used to characterize CSF metabolome. RPLC: reverse phase liquid chromatography; ESI: electrospray ionization; DDA: data-dependent acquisition; PRM: parallel reaction monitoring.

A pseudo-targeted strategy was adopted to discern the infection-related metabolic changes in CSF (Scheme 1). In the discovery stage, untargeted analysis was performed to characterize CSF metabolome of the six control and eight infection samples and select differential metabolites as potential targets. The patients with bacterial CNS infection were all diagnosed by bacterial culture together with clinical symptoms and routine biochemical items of biofluids, using the procedure shown in Figure S1. The CSF diagnostic parameters, bacterial culture results, and blood inflammatory marker quantification results of the patients and controls are summarized in Table 1. In the verification stage, targeted analysis offered reliable quantitation to test the significance of the targets. In the function prediction stage, pathway analysis related our experimental results with prior curation, broadening the biological picture regarding the intracranial bacterial infection of pediatric patients.

### 2.1. Untargeted Analysis for Metabolic Profiling of CSF from Patients with Intracranial Bacterial Infection

**Figure 1:**
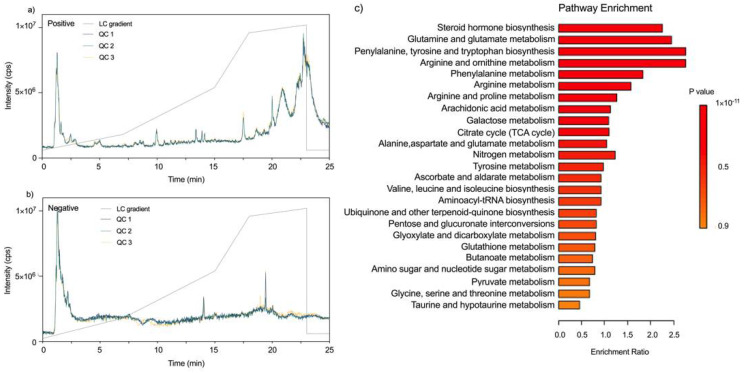
The LC-MS total ion current chromatograph (TIC) of QC samples in the positive mode (**a**) and negative mode (**b**), the dotted lines show the LC gradient in B phase percentage. (**c**) Top 25 metabolic pathways enriched in children CSF samples by untargeted metabolomic analysis.

As shown in Figure 1a,b, the total ion chromatograms (TICs) of quality control (QC) samples overlapped well in both positive and negative modes, indicating the sound reproducibility of the LC-MS system. Unsupervised PCA analyses based on the LC-MS features of patients and controls are shown in Figure S2. A total of 597 metabolites were identified by the untargeted profiling of the 14 samples (eight patients and six controls) combining the positive and negative mode based on accurate molecular weight assignment and tandem MS pattern matching, including 284 human metabolites not yet included in the current CSF metabolite library (see Dataset S1) [3,8]. Both polar metabolites (amino acids, purines, monosaccharides, etc.) and non-polar metabolites (eicosanoids, phenols, steroids, etc.) were identified (see Figure S3). With functional enrichment analysis by MetaboAnalyst, it was found that the identified metabolites covered most of the pathways known to be involved in infection pathogenesis (Figure 1c), such as glutamine and glutamate metabolism, arginine metabolism, and ascorbate and aldarate metabolism [29,31,36].

**Figure 2:**
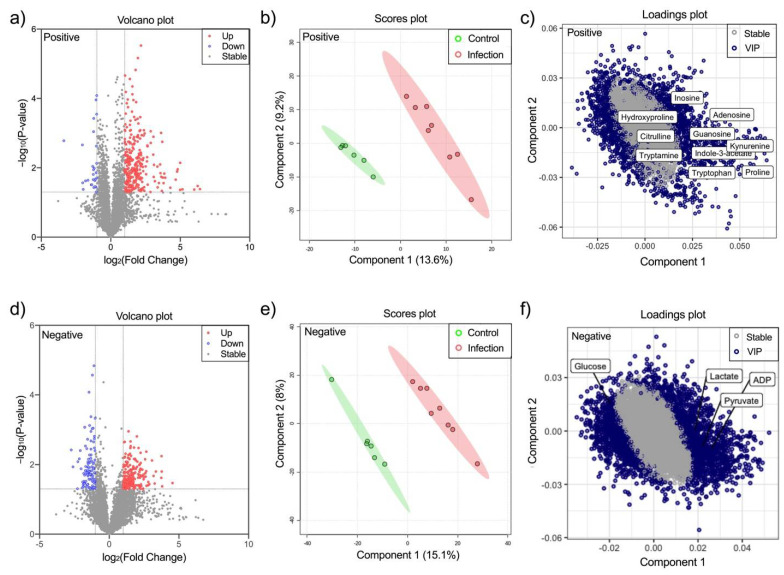
Volcano plots of CSF metabolomic features from infection samples and controls in the positive ion mode (**a**) and negative ion mode (**d**). The features up-regulated and down-regulated in the infection group are plotted in red and blue, respectively. The dotted lines show the threshold as FC = 2 and *p* value = 0.05. The PLS-DA score plots for CSF metabolomic features from infection samples and controls in the positive ion mode (**b**) and the negative ion mode (**e**). PLS-DA loading plots in the positive ion mode (**c**) and the negative ion mode (**f**).

To find out the significant metabolic features in the patients with intracranial bacterial infection compared to the control group, univariate analysis and multivariate analysis were performed on the untargeted metabolomic data acquired in positive and negative modes. Univariate analysis screened out a total of 618 significant features (fold change (FC) ≥ 2 and *p* value ≤ 0.05), among which 477 were up-regulated and 141 down-regulated (Figure 2a,d). The slightly skewed distribution suggested an increase in total metabolite concentration in the infection group. Partial least squares regression-discriminant analysis (PLS-DA) model was further used for feature selection [37]. As shown in Figure 2b,c,e,f, the model separated the two cohorts well on the feature level in both positive and negative mode. The goodness-of-fit parameters (accuracy, R^2^ and Q^2^) of the PLS-DA model is shown in Figure S4. We included features from the top five components in the positive ion mode and top two components in the negative ion mode for differential metabolites screening. Of the 4125 variable importance in the projection (VIP) features, the 618 univariately significant features were all included (Figure 2). After identification, a total of 206 differential metabolites were screened out (VIP ≥ 1) (see Figure S5, Dataset S2). It should be noted here the raw *p*-value, instead of multiple testing correction adjusted *p*-value, is used together with multivariate analysis as a cut-off for feature screening, due to the heterogeneity among biological samples and the imbalance between the large number of features and the small sample set. The screened features were then subjected to further validation by targeted analysis and pathway analysis.

### 2.2. Targeted Metabolomic Analysis for Semi-Quantitative Verification and Pathway Analysis

**Figure 3:**
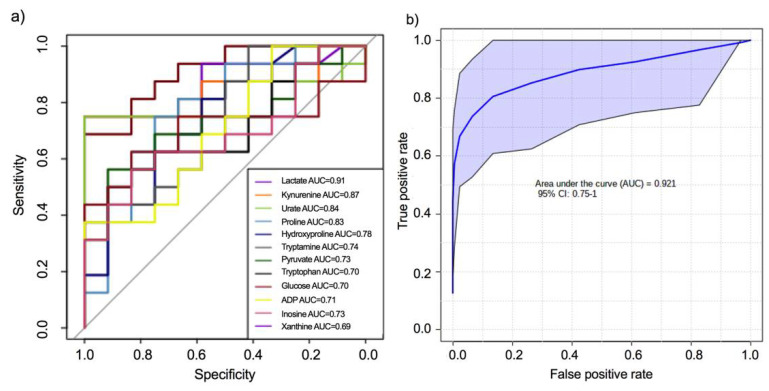
Receiver operating curves of differential metabolites verified by targeted analysis: (**a**) Univariate ROC; (**b**) random forest-based multivariate receiver operating curve combining the 12 metabolites.

**Figure 4:**
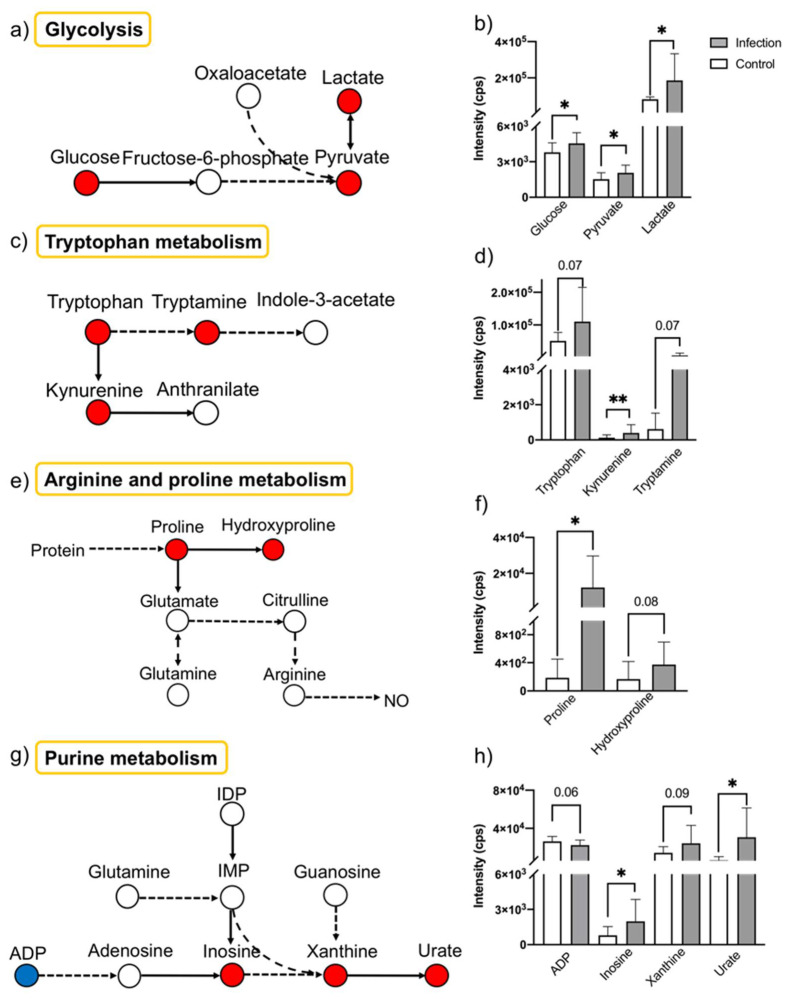
Overview of the metabolic dysregulation in glycolysis (**a**), tryptophan metabolism (**c**), arginine and proline metabolism (**e**), and purine metabolism pathways (**g**), up-regulated, stable, and down-regulated metabolites are marked in red, white, and blue, respectively; solid and dotted lines indicate direct and indirect reactions, respectively; significant intensity differences between infection and control cohorts verified by targeted metabolomic analysis (**b**,**d**,**f**,**h**), error bars represent the standard deviation, and “*” and “**” represent *p* values less than 0.05 and 0.01, respectively.

Eighteen of the differential metabolites were chosen as targets based on biological knowledge in prior publications, namely glucose, pyruvate and lactate in the glycolysis pathway, tryptophan, kynurenine, tryptamine, indole-3-acetate in the tryptophan metabolism pathway, ADP, adenosine, inosine, guanosine, xanthine, urate in the purine metabolism pathway, and proline, hydroxyproline, glutamine, arginine, citrulline in the arginine and proline metabolism pathway [19,29,30,31]. Targeted analysis was performed on the metabolites for further semi-quantification and verification (see Dataset S3). Two injections were performed on each sample. Twelve (67%) of the targets passed the verification (with *p* value < 0.1), and showed fine discriminatory potential in CV-ROC test, both univariately and multivariately (Figure 3). Among the 12 significant metabolites, the specificity of lactate, tryptophan and kynurenine has been tested across different subtypes of CNS infection in previous reports [30,38]. Pathway analysis based on the 12 significant metabolites showed the dysregulation in glycolysis, tryptophan metabolism, arginine and proline metabolism, and purine metabolism (Figure 4). The dysregulated pathways were reported to be involved in several infection-induced processes as immune response and oxidative stress [36,39].

The dysregulation in glycolysis suggests an altered energy metabolism. The elevated flux from glucose to lactate may, according to the Warburg effect, result from the aerobic glycolysis of infection-activated immune cells (Figure 4a,b) [40]. Further, the accumulation of lactate downstream plays an immunosuppressive role by down-regulating rate-limiting glycolytic enzymes upstream [41]. The increase in lactate/pyruvate ratio (see Figure S6a) also suggests that other mechanisms are involved, such as anaerobic glycolysis as a result of infection-induced hypoxia [42]. The activated tryptophan metabolism suggests different immunoregulation and neuro-damage. A higher kynurenine/tryptophan ratio in the infection group represents a higher activity of indoleamine-2,3-dioxygenase (IDO) (see Figure 4c,d, Figure S6b) [30]. The up-regulation of IDO is in response to cytokines and inflammatory signaling molecules. Activated IDO and elevated kynurenines downstream have been reported to play an immunosuppressive role [39,43]. Downstream metabolites of kynurenines also exhibit differed neurological effects: kynurenic acid, picolinic acid and nicotinamide adenine dinucleotide being neuroprotective, while quinolinic acid being neurotoxic [44]. The elevation in arginine and proline metabolism suggests a protective response to oxidative stress. The increase in proline can be attributed to the increase in CSF protein upstream (Figure 4e,f). Under infection-induced oxidative stress, proline can interact with hydroxy peroxide, generating hydroxyproline and nitroxide radicals [45]. In this way, proline helps scavenge reactive oxygen species, and complement the arginine metabolism pathway in generating nitric oxide, a molecule that plays a dual role in neuroprotection and anti-microbial defense [46]. The dysregulated purine metabolism suggests perturbed oxidative homeostasis and purinergic signaling. Elevation of xanthine and urate suggests the activation of xanthine oxidase (XO), which sustains oxidative homeostasis by generating multiple reactive species, including hydrogen peroxide and urate (Figure 4g,h) [36]. It has been reported that the up-regulation in inosine and urate are a protective response, and thus severity-related [33,47]. Additionally, the streamline from ATP to urate plays a pivotal role in purinergic signaling, forming a balance between pro-inflammation (by ATP) and anti-inflammation (by adenosine) [48].

By untargeted and targeted metabolomic analysis, we provide potential biomarkers and pathological mechanisms for CNS bacterial infection. This knowledge can further contribute to clinical diagnosis and drug development. In this pilot study, CSF from children with hydrocephalus was used as the aseptic control cohort, which does not strictly correspond to the healthy CSF. Given the ethical issues regarding the procurement of CSF from healthy children, CSF from children with hydrocephalus remains a common choice of control group to mimic sterile and less-inflammatory states [49,50,51]. Larger sample sets are desired to train a robust classification model and to validate the potential pathological pathways proposed in this paper. However, it was not easy to collect CNS bacterial infection pediatric patients. Not knowing the bacteria strains involved in postoperative infection, we focused on the *Homo sapiens* metabolic pathways instead of the bacteria metabolic pathways. Therefore, further efforts are needed to dissect the co-metabolism of host and pathogen. Moreover, while our work focused on polar metabolites, it has been reported that certain lipid classes, such as phosphatidylcholines, sphingolipids and ceramides, play an important role in CNS infection [52,53]. Multi-omics characterization of CSF, as well as peripheral blood are expected for an integrated and detailed regulatory landscape regarding CNS infection.

## 3. Materials and Methods

### 3.1. CSF Sample Collection and Patient Information

Clinical information and CSF samples were collected during lumbar puncture from hydrocephalic pediatric patients (without intracranial infection, *n* = 6) and pediatric patients of postoperative intracranial bacterial infection (*n* = 8) at the neurosurgery department of the Children’s Hospital of Shanghai, China (Table 1). Between the infection and control groups, there was no significant difference in age and gender but a significant difference in CSF protein level and CSF lactate level with a p-value cut-off of 0.05. The 6 control and 8 infection samples were used for non-targeted and targeted metabolomic analysis. The CSF samples were centrifuged at 3000 rpm under 4 °C for 15 min to remove cells. Supernatants were collected and stored in 200 μL aliquots at −80 °C before further use.

### 3.2. Chemicals and Reagents

HPLC grade acetonitrile (ACN) and methanol were purchased from Hushi Laboratorial Equipment (Shanghai, China). Analytical grade formic acid was purchased from J&K Scientific (Beijing, China), and caffeine was purchased from Sigma-Aldrich (St. Louis, MI, USA). Deionized (DI) water (18.2 MΩ∙cm) was obtained from a Milli-Q system (Millipore, Bedford, MA, USA) and used in all experiments. All chemicals were used as received without further purification.

### 3.3. Metabolite Extraction

CSF samples were thawed at room temperature. For the analysis, 100 μL of the CSF sample was thoroughly mixed with 400 μL of ACN:MeOH (1:1, *v*/*v*), followed by centrifugation at 13,000 rpm for 15 min at 4 °C. The supernatant was then lyophilized at −40 °C overnight. The dried extracts were then reconstituted in 150 μL of H_2_O:ACN:MeOH (2:1:1, *v*/*v*), sonicated for 1 min, and centrifuged at 15,000 rpm under 4 °C for 20 min. A total of 145 μL of the supernatant was transferred to an LC auto-sampler vial (CNW technologies, Shanghai, China). A total of 20 μM of caffeine was added as the internal standard for untargeted analysis in the positive ion mode. A QC sample was prepared by mixing 10 µL of each sample treated as above, which was used to check the stability of the analytical conditions.

### 3.4. LC-MS/MS Analysis

For both untargeted and targeted metabolomics, LC-MS/MS analysis was performed on a TripleTOF 4600 mass spectrometer (SCIEX, Boston, MA, USA) coupled to a LC-20AD XR high performance liquid chromatography system (Shimadzu, Kyoto, Japan). Samples of different cohorts were placed in a randomized order. One QC sample and one solvent were injected after each of the 5 CSF samples. The separation was performed using an Acquity UPLC HSS T3 column (100 × 2.1 mm, 1.8 µm, Waters, Elstree, UK) in binary gradient mode. The mobile phases in different ionization modes were as follows: (A) 0.1% (*v*/*v*) formic acid in water and (B) pure ACN in positive mode; (A) pure water and (B) pure ACN in negative mode. The flow rate was 200 μL·min^−1^. In positive mode, an optimized gradient of 0–3.5 min (B, 5–25%), 3.5–13 min (B, 25–55%), 13–18 min (B, 55–80%), 18–23 min (B, 80–85%) with a 5 min re-equilibration applied. In negative mode, an optimized gradient of 0–3.5 min (B, 2–25%), 3.5–13 min (B, 25–55%), 13–18 min (B, 55–80%), 18–23 min (B, 80–85%) with a 5 min re-equilibration applied. Column temperatures were maintained at 25 °C, and the injection volumes were set as 8 µL and 7 µL for positive and negative modes, respectively. For untargeted analysis, the mass scanning range was *m*/*z* 50–1000. The data-dependent acquisition (DDA) cycle took 1.3 s with a TOF-MS scan for 0.25 s and at most 10 MS/MS spectra acquisition for 0.1 s each. For targeted analysis, the cycle was consisted of a 0.09 s TOF-MS scan and several 0.09 s parallel reaction monitoring (PRM) channels. The precursor and product ion pairs acquired were listed in Dataset S3.

### 3.5. Statistical Analysis

For untargeted analysis, the XCMS package (https://bioconductor.org, accessed on 30 December 2020) was used to process all data files obtained by the LC-MS/MS. Univariate statistical analysis was performed with fold change (FC) = 2 and *t*-test *p* value = 0.05 as thresholds. Multivariate analysis was processed using MetaboAnalyst 5.0 (McGill University, Montréal, Quebec, Canada) [54]. Logarithm transform and pareto scaling were performed. In PLS-DA, features with a VIP value over 1 were selected as significant features.

For targeted analysis, the areas of target peaks were extracted by PeakView (SCIEX, Boston, MA, USA). T-test was performed to compare peak areas of metabolites. All experimental data were presented as means ± standard deviation (SD). Cross validation–receiver operating curve (CV-ROC) was performed by pROC RPackage (https://cran.r-project.org/web/packages/pROC/index.html, accessed on 30 July 2021) and MetaboAnalyst 5.0 (https://www.metaboanalyst.ca/, accessed on 30 July 2021) [55]. Multi-variate ROC curves were generated by Monte Carlo cross validation (MCCV). In each MCCV, 2/3 of the samples were used to evaluate the feature importance. The top important features were then used to build the random forest classification models which was validated on the 1/3 the samples that were left out. The procedure was repeated multiple times to calculate the performance and confidence interval of each model.

### 3.6. Identification and Pathway Analysis

The structural identification of metabolites was performed by the MetDNA web server (http://metdna.zhulab.cn/, accessed on 30 July 2021) and the mzmine 2 tool (http://mzmine.github.io/, accessed on 30 July 2021) [56,57]. Function enrichment analysis was carried out using MetaboAnalyst 5.0 against the *Homo sapiens* Kyoto encyclopedia of genes and genomes (KEGG) dataset (https://www.kegg.jp/, accessed on 30 July 2021) [58]. Pathway analysis was implemented on KEGG databases.

## 4. Conclusions

Combining the untargeted and targeted analysis of CSF metabolome, we identified 12 metabolites that discriminate pediatric cases of postoperative intracranial bacterial infection against control group. We also reported the dysregulation in the glycolysis, tryptophan metabolism, arginine and proline metabolism, and purine metabolism among the infection pediatric cases. These perturbed pathways depicted the metabolic changes under infection-induced oxidative stress and immune response. Characterization of the infection-induced metabolic changes could provide robust biomarkers for clinical diagnosis, novel pathways for pathological investigations, and new targets for treatment.

## Figures and Tables

**Table 1:** Patient information.

Group	Infection	Control
Age	2.1 ± 4.4 years	1.6 ± 2.2 years
Gender	62.5% male	66.7% male
CSF cell count ($\times 10^{6}$/L)	274 ± 132	6.25 ± 8.07
CSF glucose (mmol/L)	1.96 ± 1.63	2.85 ± 0.32
CSF protein (mg/L)	997 ± 562	350 ± 37
CSF lactate (mmol/L)	3.45 ± 0.08	1.50 ± 0.33
CSF bacterial isolates	75% negative 25% *S. epidermidis* positive	100% negative
Blood WBC ($\times 10^{9}$/L)	17.06 ± 6.88	9.15 ± 3.01
Blood N%	51.0 ± 17.8%	34.9 ± 14.0%
Blood CRP (mg/L)	31.0 ± 37.8	5.75 ± 3.03
Blood PCT (ng/mL)	1.29 ± 0.79	NA

WBC: white blood count; N: neutrophil; CRP: C-reactive protein; PCT: procalcitonin; *S. epidermidis*: *Staphylococcus epidermidis*.

## Data Availability

Data are available on request from the correspondence authors.

## Supplementary Materials

The following are available online, Figure S1: The workflow of CNS infection diagnosis, Figure S2: Unsupervised PCA analysis, Figure S3: Classification of identified metabolites, Figure S4: Goodness-of-fit parameters, Figure S5: Heatmaps of differential metabolites, Figure S6: Differences in the intensity ratio of downstream over upstream metabolites, Dataset S1: Metabolites identified in untargeted analysis, Dataset S2: Differential metabolites between the infection and control groups, Dataset S3: Relative quantification of 18 metabolites by targeted analysis.

## Sample Availability

Samples of the compounds are not available from the authors.
