# Supplementary material for: Metabolomic Characterization of Cerebrospinal Fluid from Intracranial Bacterial Infection Pediatric Patients: A Pilot Study
Source: Molecules. 2021 Nov 15;26(22):6871. doi: 10.3390/molecules26226871 (PMC8622478; doi:10.3390/molecules26226871)
Supplement: Supplementary file 1 [file molecules-26-06871-s001.zip › supplementary files-3/Supporting Information revised1109.pdf]

Supplementary Materials

Metabolomic Characterization of Cerebrospinal Fluid from Intracranial Bacterial Infection Pediatric Patients: A Pilot Study

Yiwen Wang [1], Yu Liu [2], Ruoping Chen [3,*], Liang Qiao [1,*]

[1] Department of Chemistry, and Shanghai Stomatological Hospital, Fudan University, Shanghai, 200000, China

[2] Department of Neurosurgery, Shanghai Children's Hospital, Shanghai Jiao Tong University, Shanghai 200062, China

[3] Department of Pediatric Neurosurgery, Xinhua Hospital, Shanghai Jiao Tong University School of Medicine, Shanghai 200092, China

* Correspondence: liang_qiao@fudan.edu.cn for LQ; rubinchen@126.com for RC

Table of contents

Figure S1 The workflow of CNS infection diagnosis. 3
Figure S2 Unsupervised PCA analysis 4
Figure S3 Classification of identified metabolites 5
Figure S4 Goodness-of-fit parameters 6
Figure S5 Heatmaps of differential metabolites 7
Figure S6 Differences in the intensity ratio of downstream over upstream metabolites 8
Dataset S1 Metabolites identified in untargeted analysis
Dataset S2 Differential metabolites between the infection and control groups
Dataset S3 Relative quantification of 18 metabolites by targeted analysis

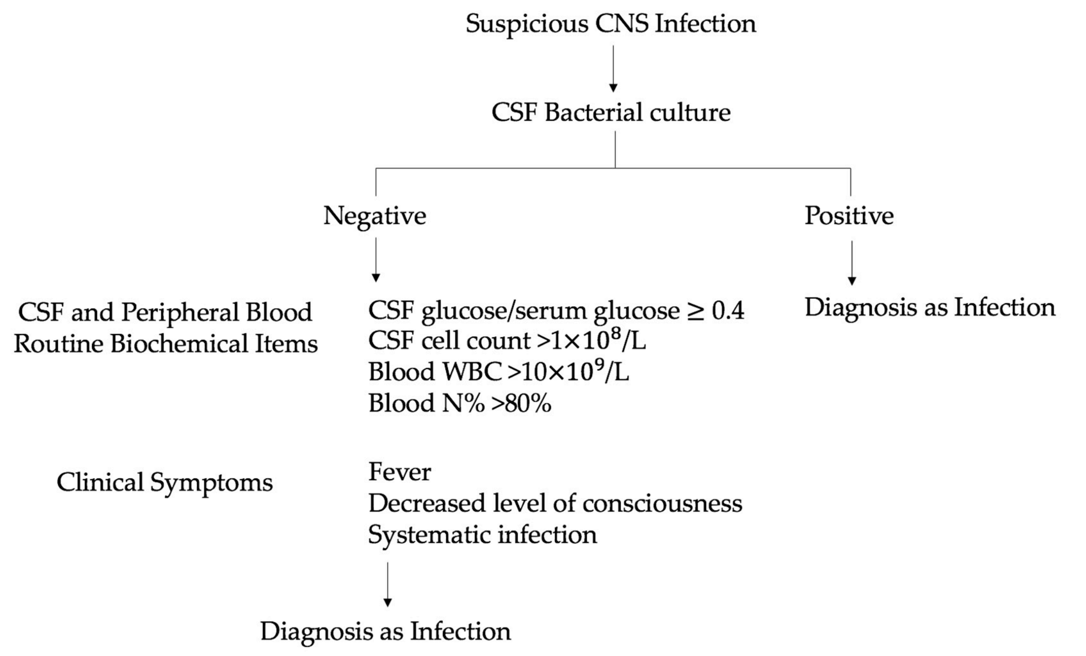

Figure S1. The workflow of CNS infection diagnosis.

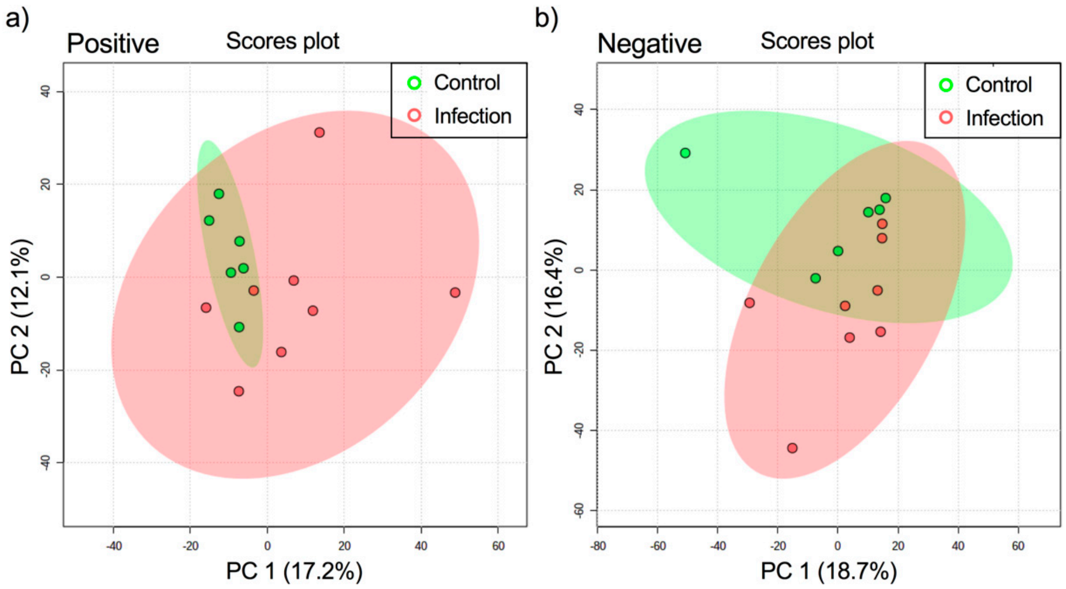

Figure S2. Unsupervised PCA analysis: PCA scores plot based on LC-MS features in the positive ion model (A) and the negative ion model (B)

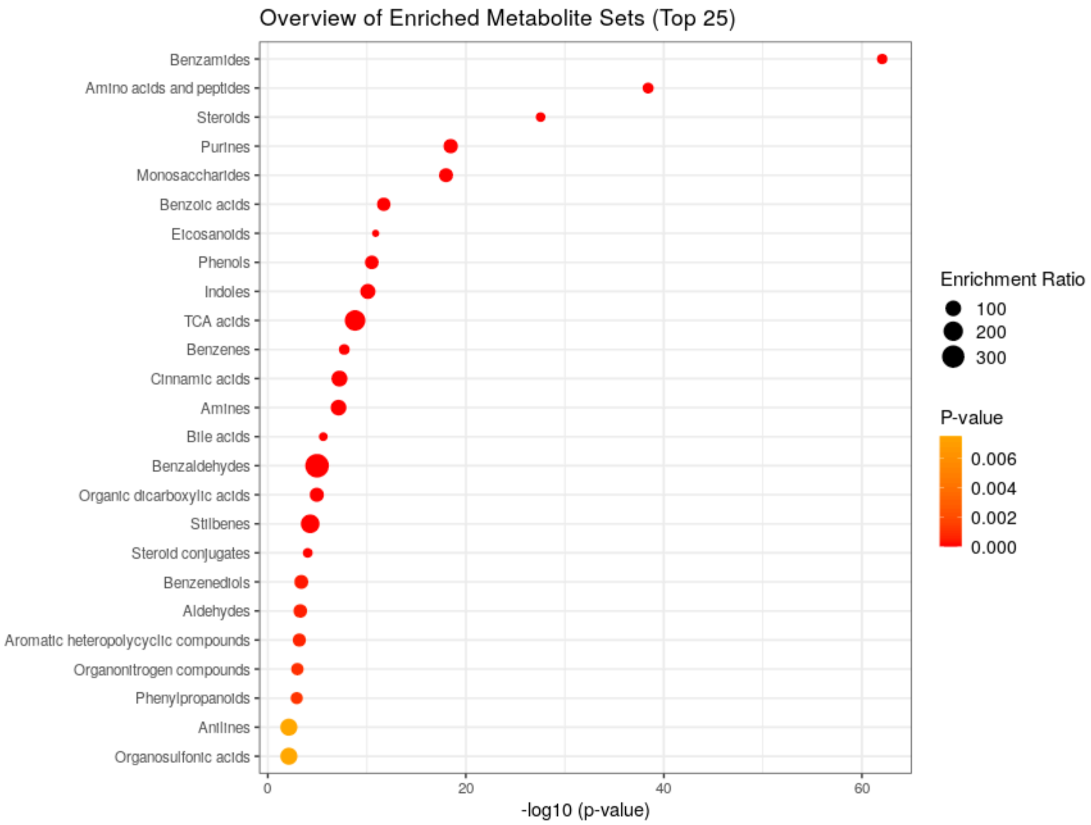

Figure S3. Metabolite classification: top 25 classes enriched from the metabolites identified by the untargeted metabolomic analysis

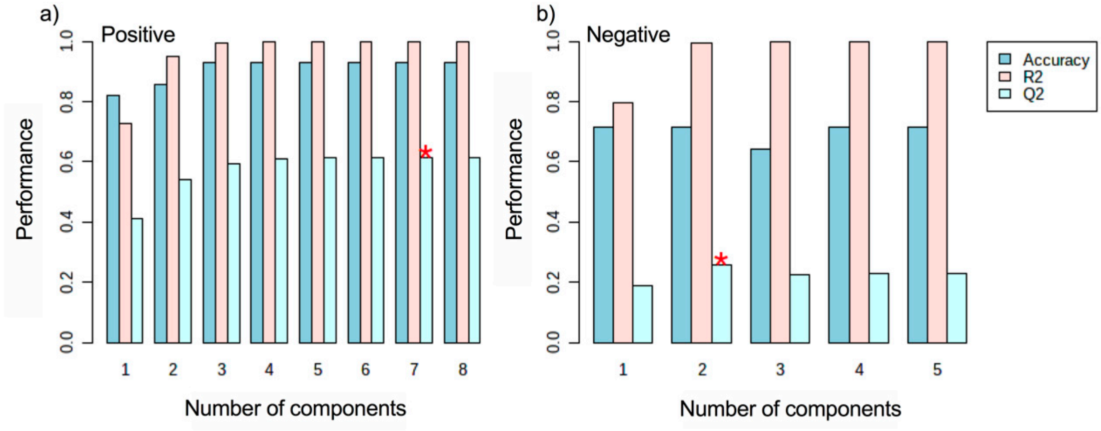

Figure S4. Goodness-of-fit parameters: R^2 and Q^2 value as a function of number of components in the PLS-DA model based on LC-MS features in the positive ion model (A) and the negative ion model (B). Asterisk indicates the highest Q^2 value.

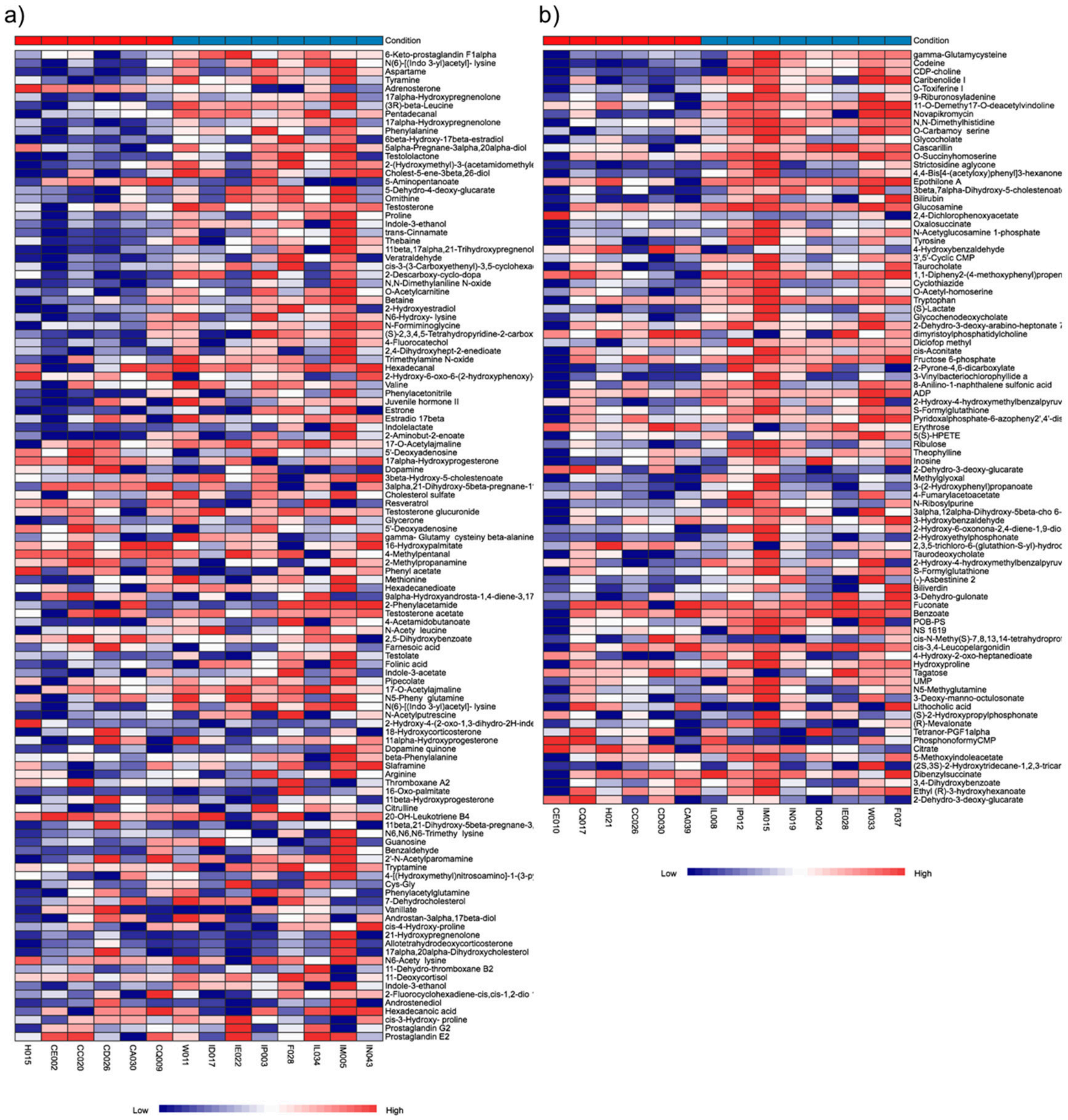

Figure S5. Heatmaps of the differential metabolites between the infection (cyan bar) and the control groups (red bar) from untargeted DDA analysis in (a) positive mode and (b) negative mode

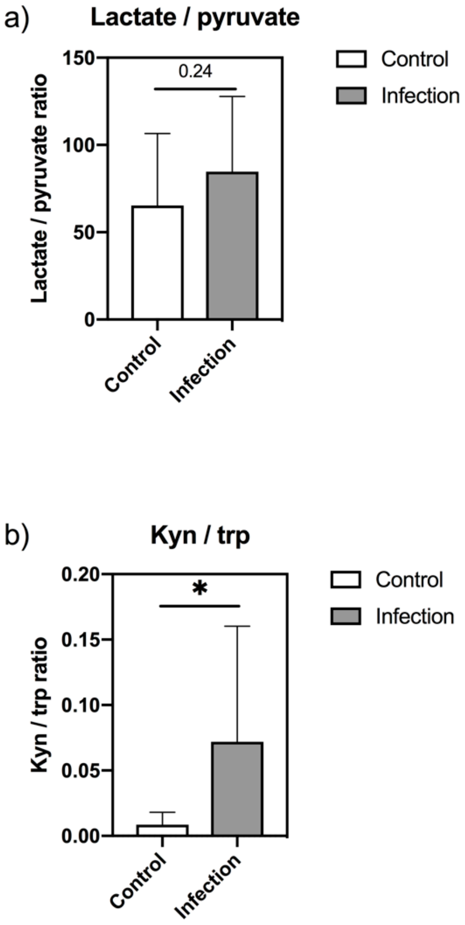

Figure S6. Differences in the intensity ratio of downstream over upstream metabolites: (a) lactate to pyruvate ratio in the glycolysis pathway, (b) kynurenine to tryptophan ratio in the tryptophan metabolism pathway.
